# Knowledge and attitudes towards complementary medicine by nursing students at a University in South Africa

**DOI:** 10.4102/hsag.v25i0.1436

**Published:** 2020-12-09

**Authors:** Renaldi van Rensburg, Radmila Razlog, Janice Pellow

**Affiliations:** 1 Department of Complementary Medicine, Faculty of Health Sciences, University of Johannesburg, Johannesburg, South Africa

**Keywords:** Complementary medicine, Nursing students, Knowledge, Attitudes, University

## Abstract

**Background:**

The increased popularity of complementary medicine has created the need for patients to receive accurate information from nurses who are front-line healthcare providers. Studies have demonstrated that patients are more likely to discuss other medication and therapy use with nurses, rather than with general practitioners or other health professionals. It is, therefore, important to determine nurses’ knowledge and attitudes towards complementary medicine.

**Aim:**

The aim of this study was to determine nursing students’ knowledge and attitudes toward the use of complementary medicine.

**Setting:**

The study was conducted with Baccalaureus Curationis (nursing) students registered at a large public university in Gauteng, South Africa.

**Methods:**

This research utilised a quantitative approach with a prospective, descriptive survey design. A convenience sample comprising registered Baccalaureus Curationis nursing students (*n* = 202) was utilized. Participants completed a 34-item, paper-and-pencil questionnaire to determine their knowledge, personal use and attitude toward complementary medicine modalities.

**Results:**

Questionnaires were completed by 126 nursing students with 119 questionnaires included for analysis. Despite a reported lack of knowledge regarding complementary medicine and limited personal use, participants had an overall positive attitude towards complementary medicine. Almost half of the participants reported enquiring about its use in history taking and were willing to refer patients to a complementary medicine practitioner.

**Conclusion:**

A positive attitude towards complementary medicine modalities might suggest a willingness from students to improve their knowledge of the various modalities and to refer to a complementary medicine practitioner when indicated.

## Introduction

Complementary medicine represents a heterogeneous group of practices and therapies that are not considered a core component of conventional medicine. According to the Global Report on Traditional and Complementary Medicine 2019, complementary medicine and its related therapies are important and underestimated health resources, with regard to their potential application in the prevention and management of lifestyle-related chronic diseases and in addressing the health needs of ageing populations (WHO 2019:5).

The increased popularity of complementary medicine has created the need for patients to receive accurate information from professional nurses who work on the front lines of mainstream medicine (Sibiya, Maharaj & Bhagwan 2017:18–19). According to Snyman (2014:44), complementary medicine use is well-established in Johannesburg, South Africa, with a good reputation for attaining and maintaining an optimal state of health amongst its users. The increasing use of complementary medicine therapies has important implications for nurses in terms of patient care and safety (Hall et al. 2017:47). Studies have demonstrated that patients are most likely to discuss alternative medication use with nurses rather than with general practitioners or other health professionals. It is, therefore, important for nurses as well as nursing students to have basic knowledge regarding the indications and contraindications for the use of complementary medicines (Hassan, Abd Hadi & Keng 2012:94–99).

Chang and Chang (2015:1466–1476) reviewed studies conducted on nurses’ knowledge, attitudes and ability to communicate risks and benefits about complementary medicine. Although 66% of the nurses reported positive attitudes about these therapies, over 77% lacked a good understanding of the risks and benefits of complementary medicine therapies. A sizable number of the nurses felt uncomfortable in discussing the use of complementary medicine with their patients. A systematic review on knowledge, attitude and use of complementary and alternative medicine among nurses by Balouchi et al. (2018:146–153) demonstrates the knowledge of complementary medicine modalities by nurses was, in fact, lower (*x* = 62.2%) than that found in the scoping review conducted by Chang and Chang (2015:1466). Current evidence demonstrates the need for nurse education programmes to integrate and strengthen complementary medicine modalities content into curricula to best serve the needs of patients receiving nursing care (Balouchi et al. 2018:146).

Alongside a growth in demand for complementary medicine, closer ties have developed between complementary medicine modality practitioners and nurses through direct integrative practice, referral or simply an acknowledgement that conventional medicine providers need to discuss concurrent use of complementary medicine with their patients (Ping 2015:1–2). The World Health Organization (WHO) asserts that education is a critical means of ensuring safe and effective practice and the use of complementary medicine. However, the curricula and level of education vary significantly within each country and across various health disciplines, which could result in potential benefits of different complementary medicine modalities not being fully recognised (WHO 2013:31).

Complementary medicine modalities are defined as treatments that evoke healing, taking into account the body–mind–spirit connection within an individual. The word ‘complementary’ conveys the idea that the modalities could be used to complement and enhance biomedical treatments (Tabish 2008:v–ix). The term ‘complementary medicine’ and its related modalities refers to a broad set of healthcare practices and treatment methods that are not part of a country’s own mainstream medicine and are not fully integrated into the dominant healthcare system but are often used interchangeably (WHO 2020). The various statutorily recognised complementary medicine modalities in South Africa include Ayurveda, Chinese medicine and acupuncture, chiropractic, homeopathy, naturopathy, osteopathy, herbal medicine, therapeutic aromatherapy, therapeutic massage therapy, therapeutic reflexology and Unani-Tibb (AHPCSA 2020).

There is little to no integration of complementary medicine curricula into healthcare practice and education in South Africa (Chitindingu, George & Gow 2014:1–5). Furthermore, it has been found that patients have insufficient information regarding complementary medicine modalities and the practice thereof. Whilst a host of reasons could explain patient decision-making factors, it is reasonable to assume that the level of knowledge as well as the attitude towards the use of complementary medicine modalities by nurses and other healthcare professionals is influential (Chitindingu et al. 2014:4).

There is abundant evidence of growing public demand for making available healthcare choices, based upon best practices, drawn from different healthcare systems. The demand for pluralism in healthcare strategies is based on a realistic assessment of the inadequacy of any single system of healthcare to address all health needs. It is probable that this assessment is responsible for the dramatic growth of interest in complementary medicine and the budding evolution of different models of complementary medicine in society (Shankar 2010:3–5). Complementary and conventional medicines should be offered alongside each other by adequately trained and well-regulated health professionals to ensure a high standard of individualised, holistic care and positive outcomes for patients. Integrating complementary medicine into the existing curricula of healthcare professionals may lead to a more open and positive attitude towards these treatment modalities (Ping 2015:1–2).

A systematic review and meta-synthesis on nurses’ attitudes towards complementary medicines and modalities by Hall and colleagues (2017:47–53) indicates that nurses have very limited education in this field and a lack of professional frameworks to assist them. There are, however, a number of barriers for nurses to support the use of complementary medicine including institutional culture and clinical context compounded by time and knowledge limitations. Numerous further studies that have focused on nurses’ perception, attitude and knowledge of complementary medicine therapies and their willingness to make use of these treatments revealed that nurses share both positive and/or negative attitudes toward certain complementary medicine modalities and do not have sufficient knowledge of these practices but demonstrate a willingness to become more knowledgeable (Balouchi et al. 2018:146; Bjerså, Victorin & Olsén 2012:1; Kavurmaci, Tan & Kavurmaci 2018:300; Lartey et al. 2019:256; Shorofi & Arbon 2017:37; Trail-Mahan, Mao & Bawel-Brinkley 2013:277).

### Theoretical framework

The theory of planned behaviour (TPB) was selected as the theoretical framework for this study. The TPB is a well-established behavioural model of human behaviour based on the concept that attitudes, subjective norms and perceived behaviour control influence one’s behaviour. Theory of planned behaviour was first proposed by Ajzen (1991) and is an extended framework of the theory of reasoned action (TRA), which has been widely used in numerous studies to understand and predict a variety of individual behaviours including health behaviours, such as adherence to prescriptions or vitamin use (Arafat & Ibrahim 2018; Passafaro, Livi & Kosic 2019). Theory of planned behaviour can be used to examine the relationship among beliefs, attitudes and behavioural intentions and actual behaviour (Dzulkipli et al. 2019). It would be useful to apply the theoretical dimensions of TPB to explain nursing students’ behavioural intention to willingly make use of complementary medicine and/or recommend it to their patients.

To date, no known studies have been conducted to determine the knowledge and attitude towards complementary medicine modalities by nursing students.

### Aim

The aim of this research was to determine the knowledge and attitudes toward complementary medicine modalities by nursing students attending a large public university in Gauteng, South Africa.

## Research methods and design

### Research population and sampling strategy

The research population comprised Baccalaureus Curationis nursing students registered at a large public university in South Africa for the 2018 academic year. Eligibility criteria included those students who were 18 years or older and who had proof of registration as a nursing student with the South African Nursing Council (SANC). Participants were recruited by means of convenience sampling (*n* = 202). The sample size was calculated with a 95% confidence interval and a margin of error of between 5% and 10% which was considered acceptable (Suresh & Chandrashekara 2012:7–13). A minimum of 65 completed questionnaires were required for analysis.

### Research design and procedure

This research study utilised a prospective, descriptive survey design, and data collection took place at a university in Gauteng. The researcher obtained permission from the Head of the Department of Nursing to address the Baccalaureus Curationis nursing students across the various years of study (years 1, 2, 3 and 4) regarding the research study. Students were provided with an information letter explaining the purpose and procedure of the study, and those who wished to participate signed a consent form.

### Instrument

A 12-item, paper-and-pencil questionnaire was used for this study. The questionnaire comprised various domains relating to the demographic profile (three items), the student nurses’ personal use (two items), their recommendations to and enquiring about use from patients (two items) as well as knowledge and attitude towards complementary medicine modalities (five items). Questions were graded in various ways; some questions required a yes/no answer, whilst the level of agreement was rated on a 3-, 4- or 5-point Likert scale, dependent on the nature of the question. Demographic questions included age, gender and year of study. Personal use of each complementary medicine was rated according to frequency (‘never’, ‘only when needed’, ‘monthly’, ‘weekly’ or ‘daily’). They were then given a list of 14 statements giving possible reasons for choosing to use complementary medicine, which they then either agreed or disagreed with. The level of knowledge of each modality was rated as ‘none’, ‘some’ or ‘a lot’, and the overall knowledge was rated on a 4-point scale. With regard to attitudes, participants were given a list of 23 statements relating to complementary medicine, and they rated their level of agreement with each one based on a 5-point scale. Participants completed the questionnaire in a private setting, and it took approximately 15–20 min to complete.

The questionnaire used in this study was adapted from a previously developed survey, which investigated the knowledge, attitude and use of complementary and integrative health strategies by nurses (Balouchi et al. 2016:121–127). Reliability of knowledge, attitude and use were verified using Cronbach’s a of 0.87, 0.75 and 0.67 respectively. Once permission was obtained to use the previously developed questionnaire, it was modified for the South African context, where only statutorily recognised complementary medicine modalities were included. Prior to the commencement of the study, a small pilot study utilising the adapted questionnaire was conducted on 10 respondents in order to refine the instrument and enhance reliability and validity. No changes were made to any questions as all the questions were deemed unambiguous and clear. The results from the pilot study were not utilised in the final analysis.

### Data analysis

Data were analysed using SPSS version 26 (IBM Support 2019). Descriptive statistics were used to evaluate each item on the questionnaire including percentages, means and standard deviations. Data were presented as frequencies and custom tables.

### Ethical considerations

Relevant permission was obtained from the university prior to conduct of the study. Participation was voluntary and participants could withdraw from the study at any point up until they submitted the questionnaire. Participants were provided with an information letter explaining the purpose and procedures of the study and were asked to sign a consent form prior to questionnaire completion. The researcher, who recruited the participants, had no direct relationship with the students, minimising the possibility of coercion. No identifiable data were requested on the questionnaire, thus ensuring anonymity. Participants completed the questionnaire independently in a private setting. Completed questionnaires were placed in a box, which was stored in the filing room at the university’s health centre. Captured data were stored in a password-protected computer. Only the researcher and supervisors had access to the data, ensuring confidentiality. There were no anticipated risks of being involved in this study. Access to results was provided upon request.

Ethical clearance number: REC-01-129-2018.

## Results

### Demographics

A total of 126 participants completed the questionnaire; however, seven questionnaires were excluded as they were incomplete, thus 119 questionnaires were analysed, yielding a response rate of 58.9%. The sample size of 119 indicated a 6% margin of error, which is considered acceptable for surveys (Suresh & Chandrashekara 2012:7–13). All participants were over 18 years of age, the majority were females (76%) and the highest percentage of them was in their third year of study (39%). See Table 1 for further details.

**TABLE 1:** Demographic data.

Gender	Number (*n*)	Percentage (%)
Female	76	90
Male	24	29
Total	100	119
**Year of study**		
First year	9	11
Second year	24	29
Third year	39	46
Fourth year	28	33

**Total**	**100**	**119**

### Personal use of complementary medicine

**FIGURE 1:**
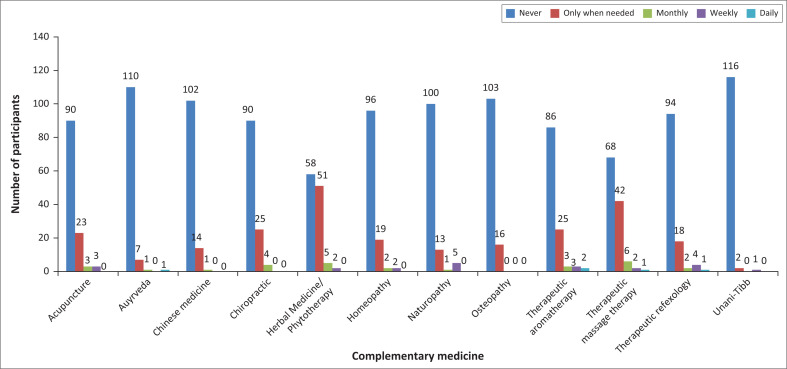
Frequency of personal use of complementary medicine modalities.

Participants rated the frequency of their personal use of each complementary medicine modality as ‘never’, ‘only when needed’, ‘monthly’, ‘weekly’ or ‘daily’. The majority of participants reported that they did not personally make use of complementary medicine as part of their healthcare (Figure 1).

Of those who did make use of complementary medicine, the main reasons given for doing so were because they felt that complementary medicine could be beneficial in helping to improve their condition (*n* = 52; 44%), maintain their health (*n* = 47; 40%) or prevent illness (*n* = 38; 32%). Less than half of the participants reported that they had easier access to it compared to conventional medicines (*n* = 49; 41%), and less than a third of the participants felt it was more cost-effective (*n* = 37; 32%), with more than a third of the participants reporting that it produced fewer adverse effects than conventional treatment options (*n* = 45; 38%). Only a small percentage of participants reported choosing complementary medicine owing to dissatisfaction with conventional medicine (*n* = 20; 17%). A number of participants felt that complementary medicine is congruent with their philosophical beliefs and way of life (*n* = 45, 38%) and also expressed a desire to exert a level of self-control over their illness (*n* = 50; 42%); whilst others felt that their health problems were not serious enough to see a healthcare practitioner (*n* = 41; 35%). Another reason participants gave for choosing complementary medicine was that it was recommended to them by friends or colleagues (*n* = 46; 39%), or their healthcare practitioner (*n* = 31; 26%).

### Enquiry of use and recommendation to patients

Participants were asked to rate how often they enquired about the use of complementary medicine during history taking. Almost half of the participants reported that they ‘always’ ask (*n* = 53; 45%), 11% (*n* = 13) ‘often’ ask, 21% (*n* = 25) ‘sometimes’ ask, 16% (*n* = 19) ‘rarely’ ask and 7.6% (*n* = 9) ‘never’ ask about complementary medicine use.

**FIGURE 2:**
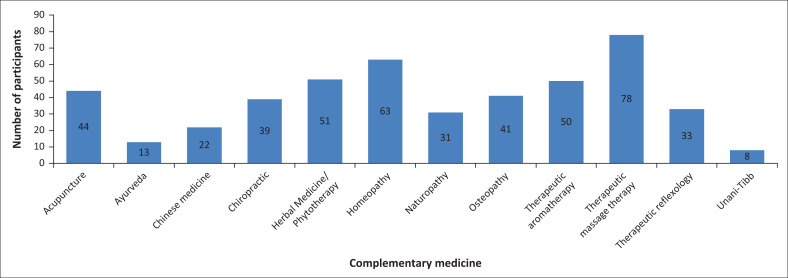
Recommendation of complementary medicine modalities to patients.

Most nursing students appeared open to recommending complementary medicine to their patients, with the most frequently recommended modalities being therapeutic massage therapy (*n* = 78; 66%), homeopathy (*n* = 63; 53%) and herbal medicine or phytotherapy (*n* = 50; 42%) in particular (Figure 2).

### Perceived level of knowledge of complementary medicine

**FIGURE 3:**
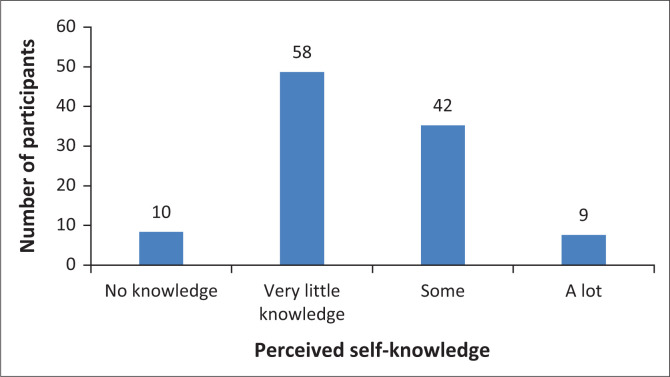
Perceived self-knowledge of complementary medicine.

Participants were asked to rate their overall perceived self-knowledge of complementary medicine modalities on a 4-point scale. As seen in Figure 3, 8.4% (*n* = 10) of participants reported having ‘no knowledge’ of the various complementary medicines modalities; whilst almost half of the respondents had ‘very little knowledge’ of complementary modalities (*n* = 58; 48%).

**FIGURE 4:**
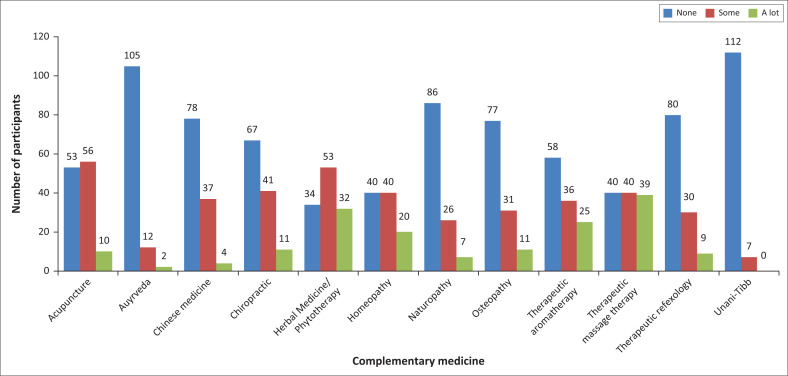
Perceived level of self-knowledge of complementary medicine modalities.

Participants were also asked to indicate their perceived level of self-knowledge regarding various complementary medicine modalities. As seen in Figure 4, most of the participants reported having no knowledge of these modalities; particularly Unani-Tibb (*n* = 112; 94%), Ayurveda (*n* = 105; 88%), naturopathy (*n* = 86; 72%), therapeutic reflexology (*n* = 80; 67%), Chinese medicine (*n* = 78; 66%) and osteopathy (*n* = 77; 65%).

### Knowledge and attitudes towards complementary medicine

Participants were given a list of 23 statements regarding complementary medicine and were asked to rate their level of agreement on a 5-point Likert-type scale where a rating of 1 = strongly disagree, 2 = disagree, 3 = neither disagree nor agree, 4 = agree and 5 = strongly agree. The results, mean score and standard deviation for each statement are presented in Table 2.

**TABLE 2:** Knowledge and attitude towards complementary medicine.

	1. Strongly disagree		2. Disagree		3. Neither disagree or agree		4. Agree		5. Strongly agree		Total		Mean score	Standard deviation
	*n*	%	*n*	%	*n*	%	*n*	%	*n*	%	*n*	%		
Complementary medicine is a branch of science.	8	6.7	8	6.7	46	38.7	30	25.2	27	22.7	119	100	3.50	1.119
Complementary medicine modalities are effective methods of treatment.	8	6.7	11	9.2	42	35.3	40	33.6	18	15.1	119	100	3.41	1.069
Complementary medicine is effective when applied together with conventional medical treatments.	16	13.4	23	19.3	33	27.7	23	193.	24	20.2	119	100	3.13	1.314
Complementary medicine modalities are effective when used separately.	21	17.6	29	24.4	33	27.7	23	19.3	13	10.9	119	100	2.82	1.249
Complementary medicine modalities can be used for the disease process.	9	7.6	20	16.8	50	42.0	26	21.8	14	11.8	119	100	3.13	1.073
Complementary medicine practices are the responsibility only of physicians.	50	42.0	32	26.9	25	21.0	7	5.9	5	4.2	119	100	2.03	1.119
Complementary medicine practices are the responsibility only of nurses.	64	53.8	29	24.4	19	16.0	4	3.4	3	2.5	119	100	1.76	1.006
Nurses have an important responsibility in complementary medicine practices.	24	20.2	22	18.5	40	33.6	15	12.6	18	15.1	119	100	2.84	1.308
Complementary medicine practices can affect medical treatment because of side effects.	15	12.6	21	17.6	32	26.9	15	12.6	36	30.3	119	100	3.30	1.393
Patients should be able to consult medical staff about complementary medicine modalities.	5	4.2	8	6.7	28	23.5	24	20.2	54	45.4	119	100	3.96	1.160
Patients who use complementary medicine modalities should inform medical staff about the issue.	3	2.5	3	2.5	22	18.5	23	68	57.1	119	100	4.26	1.012
Complementary medicine practices require a multidisciplinary approach.	7	5.9	3	2.5	22	18.5	32	26.9	55	46.2	119	100	4.05	1.134
Patients with an untreatable condition must seek complementary medicine treatment.	17	14.3	15	12.6	48	40.3	21	17.6	18	15.1	119	100	3.07	1.219
Patients are not adequately informed about complementary medicine in hospitals.	14	11.8	12	10.1	23	19.3	19	16.0	51	42.9	119	100	3.68	1.414
Patients should have the opportunity to choose between complementary medicine and conventional treatments in healthcare.	14	11.8	12	10.1	21	21.8	24	20.2	43	36.1	119	100	3.59	1.374
Conventional healthcare is too impersonal.	16	13.4	27	22.7	53	44.5	11	9.2	12	10.1	119	100	2.80	1.109
People are afraid of conventional examinations and treatment.	11	9.2	17	14.3	35	35.3	28	23.5	21	17.6	119	100	3.26	1.182
Conventional healthcare services do not meet individual’s expectations.	21	17.6	27	22.7	51	42.9	11	9.2	9	7.6	119	100	2.66	1.107
Some complementary medicine modalities are as effective as conventional treatment.	8	6.7	11	9.2	42	35.3	27	22.7	31	26.1	119	100	3.52	1.171
Complementary medicine modalities are completely safe to use.	12	10.1	14	11.8	50	42.0	16	13.4	27	22.7	119	100	3.27	1.226
Positive effects of complementary medicine are mostly because of placebo effects.	21	17.6	22	18.5	43	36.1	17	14.3	16	13.4	119	100	2.87	1.252
Complementary medicine might involve unknown risk factors for the individual’s health.	11	9.2	10	8.4	43	36.1	23	19.3	32	26.9	119	100	3.46	1.234
Surgical patients can be helped by complementary medicines.	12	10.1	16	13.4	47	39.1	30	25.2	14	11.8	119	100	3.15	1.117

**FIGURE 5:**
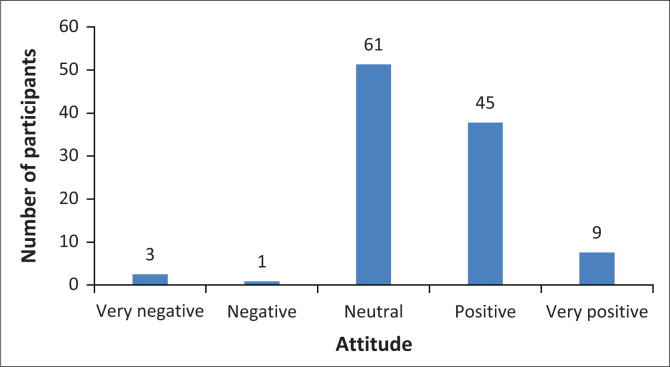
Overall attitude towards complementary medicine.

Participants were also asked to identify their overall attitude towards complementary medicine. As seen in Figure 5, slightly more than half of the respondents had a neutral (*n* = 61; 51.2%) view, with less than half having a combined ‘positive’ and ‘very positive’ view (*n* = 54; 45.4%), of complementary medicine.

## Discussion

This research study aimed to determine the knowledge of and attitude towards complementary medicine among nursing students at a large public university in Gauteng, South Africa, by means of a questionnaire. A further objective was to determine the personal use of the various complementary medicine modalities. Results demonstrated that personal use of complementary medicine was limited, with more than two thirds of the participants reporting that they had never used these modalities before. These findings are consistent with the work of Balouchi et al. (2016:121–127) who also noted minimal complementary medicine use by nursing students.

Of those participants who did make use of complementary medicine, various reasons were given as to why they chose these treatment options. Many of them felt that complementary medicine had the potential to improve and maintain their health as well as to prevent illness. Similarly, other studies have found that most nurses have a positive attitude towards the potential helpfulness of complementary medicine therapies and believe that their use might promote recovery and the healing process (Jong, Lundqvist & Jong 2015:642; Lindquist et al. 2018:63; Shorofi & Arbon 2017:37). Çamurdan and Gül (2013:350) found that nursing students prefer the use of complementary medicine in addition to conventional medical treatments to treat their own illnesses and improve their quality of life.

Dissatisfaction with conventional medicine did not appear to be a widely held motivating factor for seeking out complementary medicines. However, participants did report having relatively easy access to complementary medicine, and some felt it was more cost-effective, as suggested by Herman et al. (2012:1), and induced fewer adverse effects than conventional medicines. Whilst the majority of complementary medicine modalities are registered with medical aid schemes in South Africa (AHPCSA 2020), these forms of healthcare remain in the private sector of the healthcare system, and, as such, the general public pays significant out-of-pocket expenditure for access and use. In contrast, a systematic review of cost studies of complementary and integrative medicine showed that some of these modalities have potential cost-effectiveness, and even cost savings, across a number of populations (Herman et al. 2012:1–13). In Switzerland, where complementary medicine is integrated into the national healthcare system, a further study showed that the majority of nurses surveyed agreed that complementary medicine therapy use decreases the society’s total healthcare costs (Jong et al. 2015:642–646).

Philosophical congruency and a desire to play an active role in their own healthcare have also been identified as the motivating factors in turning to complementary medicine options. In a study conducted in the United Kingdom, researchers found that complementary medicine use appears to be driven more by patient values and beliefs than by dissatisfaction with biomedicine (Harris et al. 2012:924).

The use of complementary medicine modalities among patients has steadily increased across the globe (WHO 2019). This trend underscores the importance of open communication between the patient and the nurse regarding its use to ensure holistic nursing care, which is imperative to achieve optimum health (Hassan et al. 2012:94; Sibiya et al. 2017:18–23). A large proportion of participants reported that they do ask patients about the use of complementary medicine in their history taking. One explanation could be that nurses are required by the SANC to ‘take a thorough medical history’ to include ‘all therapies and medicines currently being used by the patient’ (SANC 2014). In contrast, a study conducted in Switzerland revealed that the majority (70%) of nurses surveyed seldom asked patients about complementary medicine use, either because of time constraints or because they did not feel it was relevant, nor their responsibility (Jong et al. 2015:642).

Whilst nurses admitted to not making personal use of complementary medicine, many reported referring patients when necessary, most notably for massage therapy, or for homeopathic or herbal treatment. The tendency for nurses to refer patients to complementary medicine practitioners has been demonstrated in previous studies, with the following modalities being highly favoured: massage therapy, aromatherapy, herbal medicine and traditional Chinese medicine (Balouchi et al. 2016:121; Jong et al. 2015:642; Pirincci et al. 2017:22).

The majority (57%) of participants reported having little to no knowledge of complementary medicine modalities. The level of knowledge of the nursing students regarding each modality was found to be highest for herbal medicine or phytotherapy, therapeutic aromatherapy and therapeutic massage therapy; and lowest for Unani-Tibb, Ayurveda, naturopathy, therapeutic reflexology, Chinese medicine and osteopathy. A study conducted at Durban University of Technology in South Africa found that only about 40% of nurses felt confident to advise patients regarding complementary medicine modalities. Lack of knowledge about complementary medicine, with little instruction in nursing programme curricula, was a primary contributing factor to a lack of confidence (Sibiya et al. 2017:18–23). This is further supported by the systematic review and meta-synthesis on nurses’ attitudes towards complementary medicines and modalities by Hall and colleagues (2017:47–56), indicating that nurses have very limited education in this field and a lack of professional framework to assist them.

With regard to nursing students’ knowledge and attitude towards complementary medicine, some interesting findings were obtained. Almost half of all participants agreed that complementary medicine is a branch of science and that it includes potentially effective forms of treatment. Many participants also agreed that patients should have the opportunity to choose between complementary medicine and conventional treatments in their healthcare. Around 65% felt that patients should be able to consult with medical staff about complementary medicine, and the majority (76%) agreed that patients should inform medical staff about complementary medicine use. Whilst most participants acknowledged that complementary medicine practices require a multidisciplinary approach, they disagreed that the responsibility lies only with medical doctors or nurses. In general, the majority of participants had either a neutral or a positive attitude towards complementary medicine. Similar results have been demonstrated in other international studies (Balouchi et al. 2016:121; Hall et al. 2017:47; Pirincci et al. 2017:22; Shorofi & Arbon 2017:37).

According to the TPB model, an individual’s intention to perform a behaviour is determined by his or her attitudes, subjective norms and perceived behavioural control; and the support for the use of this model in predicting the use of complementary medicine has been previously investigated in several studies (Furnham & Lovett 2006; Kam et al. 2010; O’Connor & White 2009; Rochelle, Shardlow & Ng 2015). In this study, it was useful to apply the theoretical dimensions of TPB to explain nursing students’ behavioural intention to willingly make use of complementary medicine and/or recommend it to their patients, and a number of influencing factors were identified. Greater complementary medicine use was associated with philosophical congruency, freedom of choice with regard to healthcare, ease of access, cost-effectiveness and perceived efficacy and safety. The relatively low level of utilisation of complementary medicine amongst nursing students is likely because of a lack of knowledge and awareness of these modalities, as reported by the majority of participants. The influence of subjective norms on behaviour was also not fully explored in this study, and this remains a limitation that needs further investigation. Regardless of the level of awareness or a positive or negative attitude towards complementary medicine, these modalities are not incorporated into the national healthcare system of South Africa, and, therefore, accessibility and affordability may also play a significant role in behavioural outcomes for some. The results of the study, therefore, somewhat concur with the theoretical underpinnings of the TPB and show that nursing students’ positive attitudes toward complementary medicine can be used to predict their behaviour with regard to its use and recommendation to patients.

There is a paucity of research related to the knowledge, attitudes and use of complementary medicines by nursing students in South Africa. This dearth of knowledge makes it difficult to compare the findings nationally. The main limitation that was identified in the study was that many of the participants did not fully understand the term ‘complementary medicine modalities’; and during the completion of the questionnaire, the researcher needed to explain the term and several of the modalities that fall under the umbrella term of complementary medicine. Future research should consider providing a definition of terms within the questionnaire to facilitate participant understanding. This issue also further highlights that students have insufficient knowledge of complementary medicines and, thus, need to be sufficiently educated in these modalities to better manage the healthcare needs of their patients.

## Conclusion

The study found that although nursing students seldom made personal use of complementary medicine, they had an overall favourable attitude towards these modalities, considering them effective, easily accessible and associated with fewer adverse effects. Most participants reported that they enquired about complementary medicine use from their patients and strongly agreed that patients should be able to consult medical staff about complementary medicine modalities. This finding highlights the need for further education on complementary medicine modalities within the nursing curriculum for nurses to safely advise patients.
